# Some results on T-stability of Picard’s iteration

**DOI:** 10.1186/s40064-016-1939-5

**Published:** 2016-03-05

**Authors:** Thokchom Chhatrajit, Yumnam Rohen

**Affiliations:** Department of Basic Sciences and Humanities, NIT Manipur, Imphal, 795001 India

**Keywords:** Partial cone, T-stable, Fixed point theorem, Picard iteration, 47H10, 54H25

## Abstract

We prove the existence and uniqueness of fixed points of T-stability for an iteration on partial cone metric space of Zamfirescu contraction. As an application, we prove a theorem for integral equation. We also give illustrative examples to verify our results.

## Background

In the year 1974, Rhoades (1974) showed that the iterative scheme converges to a fixed point of a self-mapping *f* for a particular space *X*. Rhoades (1991) provided a survey of iteration procedures that have been used to obtain fixed points for maps satisfying a variety of contractive conditions. Rhoades (1990) showed that several iteration procedures are *T*-stable for maps satisfying a fairly general contractive condition. Liu (1995) introduced the concept of the Ishikawa iteration process with errors and obtained a fixed point of the Lipschitzian local strictly pseudo-contractive mapping. Yousefi (2012) proved an iteration procedure in cone metric spaces. Rhoades and Soltuz (2006) showed that *T*-stability of Mann and Ishikawa iterations are equivalent. Qing and Rhoades (2008) established a general result for the stability of Picard’s iteration. Asadi et al. (2009) investigated the *T*-stability of Picard’s iteration procedures in cone metric spaces and gave an application. Saadati et al. (2009) showed that the variational iteration method for solving integral equations is T-stable. Recently, iteration scheme is extended to some other spaces. It is suitable for mathematician to consider *T*-stability for new iterations problems (see Saipriya et al. 2015; Kang et al. 2015; Yao et al. 2015; Haddadia 2014; Okeke and Olaleru 2014).

Let us consider a self mapping $T:X\rightarrow X$ in a complete partial cone metric space (*X*, *p*). Further, let $F_{T}=\{x \in X: Tx=x\}$ is the set of fixed points of *T*. In complete metric space, the Picard iteration process $\{x_{n}\}$ is defined by

1 $$x_{n+1}=Tx_{n}, \quad n=0,1,2,\ldots$$

It has been used to approximate the fixed points of mappings satisfying the contractive condition

2 $$d(Tx,Ty)\le \alpha d(x,y), \quad \forall x,y \in E, \alpha \in [0,1)$$

over the years by many authors. The above contractive condition (2) is called *Banach’s contraction condition*.

We shall state some of the iteration process generalising (1) as follows:

For $x_{0} \in E$, the sequence $\{x_{n}\}_{n=0}^{\infty }$ defined by

3 $$x_{n+1}=(1-\alpha _{n})x_{n}+\alpha _{n}Tx_{n}, \quad n=0,1,2,\ldots$$

where $\{\alpha _{n}\}_{n=0}^{\infty }\subset [0,1]$, is called the Mann iteration process.

For $x_{0} \in E$, the sequence $\{x_{n}\}_{n=0}^{\infty }$ defined by

4 $$\left. \begin{array}{ll} &x_{n+1}= (1-\alpha _{n})x_{n}+\alpha _{n}Tz_{n} \\ &z_{n}= (1-\beta _{n})x_{n}+\beta _{n}Tx_{n} \end{array} \right\} \quad n=0,1,\ldots$$

where $\{\alpha _{n}\}_{n=0}^{\infty }$ and $\{\beta _{n}\}_{n=0}^{\infty }$ are sequences in [0, 1] called the Ishikawa iteration process.

Kannan (1968) established an extension of the Banach’s fixed point theorem by using the following contractive definition:

For a self map *T*, there exists $\beta \in (0,\frac{1}{2})$ such that

5 $$d(Tx,Ty)\le \beta [d(x,Tx)+d(y,Ty)],\quad \forall x,y \in E.$$

Chatterjea (1972) gave the following contractive condition:

For a selfmap *T*, there exists $\gamma \in (0,\frac{1}{2})$ such that

6 $$d(Tx,Ty)\le \gamma [d(x,Ty)+d(y,Tx)],\quad \forall x,y \in E$$

Zamfirescu (1972) established the generalisation of the Banach’s fixed point theorem by combining (2), (5) and (6). For a mapping $T:E\rightarrow E$, there exist real numbers $\alpha ,\beta ,\gamma$ satisfying $0\le \alpha <1, 0\le \beta <\frac{1}{2},0\le \gamma <\frac{1}{2}$ respectively such that for each $x,y \in E$, at least one of the following is true:

1. $$d(Tx,Ty)\le \alpha d(x,y)$$
2. $$d(Tx,Ty)\le \beta [d(x,Tx)+d(y,Ty)]$$
3. $$d(Tx,Ty)\le \gamma [d(x,Ty)+d(y,Tx)]$$

Then the mapping $T:E\rightarrow E$ satisfying all above three conditions is called a Zamfirescu operator. Any mapping satisfying the above condition 2 is called a Kannan mapping while the mapping satisfying the above condition 3 is called a Chatterjea operator. Results on stability and T-stability of Picard iteration using the contractive conditions can be found in Rhoades (1974, 1990, 1991), Liu (1995), Yousefi (2012), Rhoades and Soltuz (2006), Qing and Rhoades (2008), Asadi et al. (2009), Saadati et al. (2009), Olatinwo (2008) and references there in.

Huang and Zhang (2007) obtained a generalisation of metric space by introducing the concept of cone metric space. They used an ordered Banach space in place of set of real numbers in metric space. They also obtained some fixed point theorems in this space for mappings satisfying various types of contractive conditions. Some results on cone metric space can be found in Singh and Singh (2014), Singh and Singh (2015), Singh and Sing (2014), Singh (2014) and references there in.

Let *E* be a real Banach space. A subset *P* of *E* is called a cone if

1. *P* is closed, non-empty and $P \ne {0}$
2. $$a,b \in {\mathbb {R}}, a,b\ge 0$$ and $$x,y \in P$$ imply $$ax+by \in P$$
3. $$P\cap (-P)={0}$$.

Given a cone $P\subset E$ we define the partial ordering ≤ with respect to *P* by *x* ≤ *y* if and only if $y-x \in P$. We write $x<y$ to denote that $x\le y$ but $x \ne y$, while $x\ll y$ will stand for $y-x \in int.P$(interior of *P*).

There are two kinds of cone. They are normal cone and non-normal cone. A cone $P\subset E$ is normal if there is a number $K> 0$ such that for all $x,y \in P$, $0\le x \le y \Rightarrow \parallel x\parallel \le K\parallel y\parallel$. In other words if $x_{n}\le y_{n}\le z_{n}$ and $lim_{n\rightarrow \infty }x_{n}=lim_{n\rightarrow \infty }z_{n}= x$ imply $lim_{n\rightarrow \infty }y_{n}=x$. Also, a cone $P\subset E$ is regular if every increasing sequence which is bounded above is convergent.

The aim of this paper is to show the existence and uniqueness of fixed points of T-stability for an iteration on partial cone metric space under Zamfirescu contraction. We give an application in integral equation. We also give illustrative examples that verifies our results.

We have the following basic definitions:

### **Definition 1**

(Huang and Zhang 2007) Let *X* be a nonempty set. Suppose the mapping $d:X\times X\rightarrow E$ satisfies the following conditions:

1. $0< d(x,y)$ for all $x,y \in X$ and $d(x,y)=0$ iff $x=y$.
2. $$d(x,y)=d(y,x)$$ for all $$x,y \in X$$.
3. $$d(x,y)\le d(x,z)+d(z,y)$$ for all $$x,y,z \in X$$.

Then *d* is called a cone metric on *X* and (*X*, *d*) is called a cone metric space.

### **Definition 2**

(Sonmez 2011) A partial cone metric space on a non-empty set *X* is a function $p:X \times X\rightarrow E$ such that for all $x,y,z \in X$

1. $x=y$ if and only if $p(x,x)=p(x,y)=p(y,y)$,
2. $$0\le p(x,x)\le p(x,y)$$,
3. $$p(x,y)=p(y,x)$$,
4. $$p(x,y)\le p(x,z)+p(z,y)-p(z,z)$$.

Then the pair (*x*, *p*) such that *X* is non-empty set and *p* is a partial cone metric on *X* is called a partial cone metric space. We know that if *p*(*x*, *y*) = 0, then *x* = *y*. But if *x* = *y*, then *p*(*x*, *y*) may not be 0.

A cone metric space is a partial cone metric space. But there are partial cone metric space which are not cone metric space. The following example verifies the statement.

### *Example 3*

(Sonmez 2011) Let $E={\mathbb {R}}^{2},P=\{(x,y) \in E: x,y \ge 0\}$, $X={\mathbb {R}}^{+}$ and $p:X \times X\rightarrow E$ defined by $p(x,y)=(max.\{x,y\},\alpha max.\{x,y\})$ where $\alpha \ge 0$ is a constant. Then (*X*, *p*) is a partial cone metric space which is not a cone metric space.

### **Definition 4**

(Sonmez 2011) Let (*X*, *p*) be a partial cone metric space. Let $\{x_{n}\}$ be a sequence in *X* and $x \in X$. Then $\{x_{n}\}$ is said to be convergent to *x* and *x* is called a limit of $\{x_{n}\}$ if

$$\lim _{n\rightarrow \infty }p(x_{n},x)=\lim _{n\rightarrow \infty }p(x_{n},x_{n})=p(x,x).$$

### **Definition 5**

(Sonmez 2011) Let (*X*, *p*) be a partial cone metric space. Let $\{x_{n}\}$ be a sequence in *X* and $x \in X$. Then $\{x_{n}\}$ is said to be Cauchy sequence if there exists an $a \in P$ such that for every $\epsilon > 0$ there is *N* such that $\parallel p(x_{n},x_{m})-a\parallel < \epsilon$ for all $n,m > N$.

### **Definition 6**

(Sonmez 2011) A partial cone metric space (*X*, *p*) is said to be complete if and only if every Cauchy sequence in *X* is convergent.

### **Definition 7**

(Olatinwo 2008) Let (*X*, *d*) be a complete metric space, $T:X\rightarrow X$ a selfmap of *X*. Suppose that $F_{T}=\{p \in E:Tp=p\}$ is the set of fixed points of *T*. Let $\{x_{n}\}_{n=0}^{\infty }\subset E$ be the sequence generated by an iteration procedure involving *T* which is defined by $x_{n+1}=f(T,x_{n}),n=0,1,2\dots$ where $x_{0}\in X$ is the initial approximation and *f* is some function. Suppose $\{x_{n}\}_{n=0}^{\infty }$ converges to fixed point *p* of *T*. Let $\{y_{n}\}_{n=0}^{\infty }\subset X$ and set

$$\epsilon = d(y_{n+1},f(T,y_{n})),\quad n=0,1,2,\ldots$$

Then, the iteration procedure is said to be *T*-stable or stable with respect to *T* if and only if $\lim _{n\rightarrow \infty }\epsilon _{n}=0$ implies $\lim _{n\rightarrow \infty }y_{n}=p$.

### *Remark 8*

(Olatinwo 2008) Since the metric space is induced by the norm, we have

$$\epsilon _{n}=\Vert y_{n+1}-f(T,y_{n})\Vert ,\quad n=0,1,2,\ldots$$

in place of

$$\epsilon _{n}=d(y_{n+1},f(T,y_{n})),\quad n=0,1,2,\ldots$$

in the definition of stability whenever we are working in normed linear space or Banach space.

## Main results

In this section we establish iteration procedure in partial cone metric spaces. This is to stretch out some recent results of T-stability. Let (*X*, *p*) be a partial cone metric space. Let $\{T_{n}\}_{n}$ be a sequence of self maps of *X* with $\bigcap _{n}F(T_{n})\ne \phi$. Let $x_{0}$ be a point of *X* and posit that $y_{n+1}=F(T_{n},y_{n})$ is an iteration procedure involving $\{T_{n}\}_{n}$, which gives a sequence $\{y_{n}\}$ of points from X.

In general, such a sequence $\{z_{n}\}$ can be acquired in the following way. Let $y_{0}$ be a point in *X*. Put $y_{n+1}=f(T_{n},y_{n})$. Let $y_{0}=z_{0}$. Now, $y_{1}=f(T_{0},y_{0})$. Because of rounding or in the function $T_{0}$, a new value $z_{1}$ approximately equal to $y_{0}$ might be procured in place of $f(T_{0},y_{0})$. Then to approximate $z_{1}$, the value $f(T_{1},y_{1})$ is determined to furnish $z_{2}$, approximation of $f(T_{1},z_{1})$. This computation is persisted to obtain $\{z_{n}\}$ as an approximate sequence of $\{y_{n}\}$.

### **Definition 9**

The iteration $y_{n+1}=F(T_{n},y_{n})$ is said to be $\{T_{n}\}$-semistable (or semistable) with respect to $\{T_{n}\}$ if $\{y_{n}\}$ converges to a fixed point *q* in $\bigcap _{n}F(T_{n})\ne \phi$ and whenever $\{z_{n}\}$ is a sequence in *X* with $lim_{n\rightarrow \infty }p(y_{n},f(T_{n},y_{n}))=0$ and $p(y_{n},f(T_{n},z_{n}))=o(t_{n})$ for some sequence $t_{n}\subset {\mathbb {R}}^{+}$, then we have $z_{n}\rightarrow z$.

### **Definition 10**

The iteration $y_{n+1}=F(T_{n},y_{n})$ is said to be $\{T_{n}\}$ stable(or stable) with respect to $\{T_{n}\}$ if $\{z_{n}\}$ converges to a fixed point *q* in $\bigcap _{n}F(T_{n})\ne \phi$ and whenever $\{z_{n}\}$ is a sequence in *X* with $lim_{n\rightarrow \infty }p(y_{n},f(T_{n},z_{n}))=0$, then we have $z_{n}\rightarrow z$.

### *Remark 11*

$T_{n} = T$ for all n that gives the definition of T-stability.

### **Theorem 12**

*Let (X, p) be a complete partial cone metric space. Let P be a normal cone with normal constant K and*$T:X\rightarrow X$*with*$F(T)\ne \phi$*. If there exists*$c \in (0,\frac{1}{2})$*such that*$p(Tx,Ty)\le c p(x,y)$*for all*$x,y \in X$*and*$u \in F(T)$*and in addition, whenever*$\{y_{n}\}$*is a sequence with*$p(y_{n},Ty_{n})\rightarrow 0$*as*$n\rightarrow \infty$*, then Picard iteration is T-stable.*

### *Proof*

Let $\{y_{n}\} \subseteq X, \epsilon _{n}=p(y_{n+1},Ty_{n})$ and $\epsilon _{n} \rightarrow 0$ as $n\rightarrow \infty$. Then for any $n \in {\mathbb {N}}$, we have

$$\begin{aligned} p(y_{n+1},u)&\le p(y_{n+1},Tx_{n})+p(Tx_{n},u)-p(Tx_{n},Tx_{n})\\ \Rightarrow p(y_{n+1},u)&\le p(Ty_{n+1},T^{n+1}x_{0})+ p(T^{n+1}x_{0},u)-p(T^{n+1}x_{0},T^{n+1}x_{0})\\ \Rightarrow p(y_{n+1},u)&\le c p(y_{n},T^{n}x_{0})+p(T^{n+1}x_{0},u)\\ \therefore \parallel p(y_{n+1},u) \parallel& \le K c\parallel p(y_{n},T^{n}x_{0}) \parallel + \parallel p(T^{n+1}x_{0},u) \parallel \rightarrow 0 \end{aligned}$$

Hence, $p(y_{n+1},u) =0$. But since $p(Ty_{n+1},Ty_{n+1})\le c p(y_{n+1},y_{n+1})=0$. We have that $p(Ty_{n+1},Ty_{n+1})=p(Ty_{n+1},u)=p(u,u)=0$. This implies that $Ty_{n}=u$. Therefore, $Lim_{n\rightarrow \infty }y_{n}=q$.

For uniqueness: Let *v* be another fixed point of *T*, then

$$p(u,v)=p(Tu,Tv)\le cp(u,v)$$

Since $c <1$ we have $p(u,v)=p(u,u)=p(v,v)$. Hence $u=v$.

Thus the fixed point of *T* is unique. $\square$

### **Theorem 13**

*Let (X, p) be a complete partial cone metric space. Let P be a normal cone with normal constant K and*$T:X\rightarrow X$*with*$F(T)\ne \phi .$*If there exists*$c \in (0,\frac{1}{2})$*such that*$p(Tx,Ty)\le c(p(Tx,x)+p(Ty,y))$*for all*$x,y \in X$*and*$u \in F(T)$*and in addition, whenever*$\{y_{n}\}$*is a sequence with*$p(y_{n},Ty_{n})\rightarrow 0$*as*$n\rightarrow \infty ,$*then Picard iteration is T-stable.*

### *Proof*

Let $\{y_{n}\} \subseteq X, \epsilon _{n}=p(y_{n+1},Ty_{n})$ and $\epsilon _{n} \rightarrow 0$ as $n\rightarrow \infty$. Then for any $n \in {\mathbb {N}}$, we have

$$\begin{aligned} p(y_{n+1},u)&\le p(Ty_{n+1},Tx_{n})+p(Tx_{n},u)-p(Tx_{n},Tx_{n})\\&\le c [p(Ty_{n+1},x_{n})+p(Tx_{n},x_{n})]+p(x_{n+1},u)\\&\le K \frac{1}{1-c}(c\parallel p(x_{n+1},x_{n})\parallel +\parallel p(x_{n+1},u)\parallel )\rightarrow 0. \end{aligned}$$

Hence, $p(Ty_{n+1},u)=0$. But since

$$p(Ty_{n+1},Ty_{n+1})\le c[p(Ty_{n+1},u)+p(Ty_{n+1},q)]=2cp(Tx_{n},u)=0.$$

We have that $p(Ty_{n+1},Ty_{n+1})=p(Ty_{n+1},u)=p(u,u)=0$. This implies that $Ty_{n+1}=u$.

For uniqueness: Let *v* be another fixed point of *T*, then

$$p(u,v)=p(Tu,Tv)\le c[p(Tu,v)+p(Tv,v)]=0.$$

Hence $p(u,v)=p(u,u)=p(v,v)=0$. We get $u=v$.

Thus the fixed point of *T* is unique. $\square$

### **Theorem 14**

*Let (X, p) be a complete partial cone metric space. Let P be a normal cone with normal constant K and*$T:X\rightarrow X$*with*$F(T)\ne \phi$*. If there exists*$c \in (0,\frac{1}{2})$*such that*$p(Tx,Ty)\le c(p(x,Ty)+p(y,Tx))$*for all*$x,y \in X$*and*$u \in F(T)$*and in addition, whenever*$\{y_{n}\}$*is a sequence with*$p(y_{n},Ty_{n})\rightarrow 0$*as*$n\rightarrow \infty$*, then Picard iteration is T-stable.*

### *Proof*

Let $\{y_{n}\} \subseteq X, \epsilon _{n}=p(y_{n+1},Ty_{n})$ and $\epsilon _{n} \rightarrow 0$ as $n\rightarrow \infty$. Then for any $n \in {\mathbb {N}}$, we have

$$\begin{aligned} p(y_{n+1},u)&\le p(Ty_{n+1},Tx_{n})+p(Tx_{n},u)-p(Tx_{n},Tx_{n})\\&\le c [p(Ty_{n+1},Tx_{n})+p(x_{n},Ty_{n})]+p(x_{n+1},u)\\&\le K \frac{1}{1-c}(c\parallel p(y_{n+1},x_{n+1})\parallel +\parallel p(x_{n+1},u)\parallel )\\&\le K \frac{1}{1-c}(c\parallel p(y_{n+1},u)\parallel +\parallel p(x_{n+1},u)\parallel )\rightarrow 0 \end{aligned}$$

Hence, $p(Ty_{n+1},u)=0$. But since

$$p(Ty_{n+1},Ty_{n+1})\le c[p(Ty_{n+1},u)+p(Ty_{n+1},q)]=2cp(Tx_{n},u)=0.$$

We have that $p(Ty_{n+1},Ty_{n+1})=p(Ty_{n+1},u)=p(u,u)=0$. This implies that $Ty_{n+1}=u$.

For uniqueness: Let *v* be another fixed point of *T*, then

$$p(u,v)=p(Tu,Tv)\le c[p(u,Tv)+p(v,Tu)]=0$$

Hence $$p(u,v)=p(u,u)=p(v,v)=0$$.

We get $u=v$. Thus the fixed point of *T* is unique. $\square$

### *Example 15*

Let $X = [0,\infty )$ and let *p* be the partial cone metric on *X* defined by $p(x,y)=\,\mid x-y\mid$. Let $T:X\rightarrow X$ such that

$$Tx=\left\{ \begin{array}{ll} 1 &\quad \hbox {if }\; x \in \{0,1\}\cup \left[ \frac{1}{2n+1},\frac{1}{2n}\right) \\ n &\quad \hbox {if }\; x \in \left[ \frac{1}{2n},\frac{1}{2n-1}\right) ,n\ge 1 \\ \frac{1}{n} &\quad \hbox {if }\;x \in (n-1,n], n\ge 2 \end{array} \right.$$

Then $p(Tx,1)\le p(x, Tx)$ for each $x \in [0,\infty )$. If $Tx=1$, then the inequality of the Theorem 12 is true. If $x \in [\frac{1}{2n},\frac{1}{2n-1}),n\ge 1$, then $Tx=n$ and

$$p(Tx,1)=n-1\le \frac{n-1}{2n-1}< n-x = p(x, Tx).$$

If $x \in (n-1,n]), n\ge 2$, then $Tx=\frac{1}{n}$ and

$$p(Tx,1)=1-\frac{1}{n}<\frac{x-1}{n}=p(x, Tx).$$

for each $x \in X$, where $q=1 \in F(T)$ and It is easy to see that Picard iteration $x_{n+1} = Tx_{n}$ converges to 1 for every $x_{0} \in X$. Let $y_{2n}=\frac{1}{2n}, y_{2n+1}=\frac{1}{4n+4}, n\ge 1$. Then

$$p(y_{2n+1}, Ty_{2n}) = \frac{1}{2n}- \frac{1}{(4n + 4)} = \frac{(n + 2)}{[4n(n + 1)]}$$

and

$$p(y_{2n+2}, Ty_{2n+1})=2n+2-2n-2=0,$$

so $$p(y_{n+1}, Ty_{n}) \rightarrow 0$$.

## An application

### **Theorem 16**

*Let X* = *C*[0, 1], ${\mathbb {R}}$*with*$\parallel f \parallel _{\infty }\,=Sup_{0\le x\le 1} \mid f(x)\mid$*for*$f \in X$*and let*$T:X\rightarrow X$*defined by*$Tf(x)=\int ^{1}_{0}F(x,f(t))dt$*where*

1. $F:[0,1]\times {\mathbb {R}}\rightarrow {\mathbb {R}}$*is a continuous function.*
2. *The partial derivative*$F_{y}$ of *Fwith respect to y exists and*$\mid F_{y}(x,y)\mid \le c$*for some*$c \in [0,1)$
3. *For every real number*$0\le a< 1$*one has*$ax\le F(x, ay)$*for every*$x, y \in [0,1]$

*Let*$P= \{(x,y)\in {\mathbb {R}}^{2}: x,y \ge 0\}$*be a normal cone and (X, p) the complete partial cone metric space defined*$p(f,g)=(\parallel f- g\parallel _{\infty },\alpha \parallel f-g\parallel _{\infty })$*where*$\alpha \ge 0$*. Then Picard’s iteration is T-stable if*$0\le c \le \frac{1}{2}$.

### *Example 17*

Let $F(x,y)=\frac{x+y}{4}$. Then *F* satisfies of Theorem 16 if $0\le c <1$. Let $T:X \rightarrow X$ be a self-map defined by $Tf(x)=x+(\frac{1}{4})+\int ^{1}_{0}f(t)dt$. Then *T* has unique fixed point and Picard’s iteration is *T*-stable.

## Conclusion

We extend and prove the T-stability of Picard’s iteration satisfying Zamfirescu contraction in partial cone metric space. Our results are more general than that of the results of metric and cone metric spaces. This result can be extended to other spaces.
